# Crystal structure of *N*,*N*′-[(ethane-1,2-di­yl)bis­(aza­nediylcarbono­thio­yl)]bis­(benzamide)

**DOI:** 10.1107/S205698901900495X

**Published:** 2019-04-18

**Authors:** Issa Samb, Nango Gaye, Rokhaya Sylla-Gueye, Elhadj Ibrahima Thiam, Mohamed Gaye, Pascal Retailleau

**Affiliations:** a Département de Chimie, UFR SATIC, Université Alioune Diop, Bambey, Senegal; b Département de Chimie, Faculté des Sciences et Techniques, Université Cheik Anta Diop, Dakar, Senegal; c Institut de Chimie des Substances Naturelles, CNRS UPR 2301, Université Paris-Sud, Université Paris-Saclay, 1 av. de la Terrasse, 91198 Gif-sur-Yvette, France

**Keywords:** crystal structure, thio­urea, ethyl­enedi­amine, benzoyl­thio­ureido

## Abstract

The reaction of benzoyl chloride and ethyl­endi­amine in the presence of potassium thio­cyanate yielded a white solid, C_18_H_18_N_4_O_2_S_2_, which consists of two benzoyl­thio­ureido moieties connected by an ethyl­ene chain. The asymmetric unit consists of one half of the mol­ecule, the complete mol­ecule being generated by crystallographic inversion symmetry. Both thio­urea moieties are in a *trans* conformation. An intra­molecular N—H⋯O hydrogen bond occurs. In the crystal, C—H⋯S and C—H⋯O hydrogen bonds link the molecules, forming layers parallel to the *ac* plane.

## Abstract 2

A new symmetrical thio­carbonohydrazone derivative with two similar benzoyl­thio­ureido functional groups has been prepared and characterized.

## Chemical context   

Thio­urea derivatives have been successfully used in the extraction of some transition metals (*i.e.* Cu^II^, Ni^II^ and Co^II^) from acidic media. Thio­urea derivatives have also been shown to possess anti­bacterial, anti­fungal, anti­tubercular, anti­thyroid and insecticidal properties (Arslan *et al.*, 2004 ▸; Cunha *et al.*, 2007 ▸). The structures of several types of thio­urea derivatives and its metal complexes have been determined in recent decades. These compounds possess two arms which can act as a tetra­dentate ligand coordinating through the S atom and the benzoyl O atom of each arm. Urea and thio­urea derivatives can behave as catalysts through double inter­action by hydrogen bonding with the substrate (Sigman & Jacobsen, 1998 ▸; Cortes-Clerget *et al.*, 2016 ▸). Thio­urea derivatives with alkyl bridges can adopt diverse conformations (Thiam *et al.*, 2008 ▸; Pansuriya *et al.*, 2011 ▸). We have recently begun to examine the coordination behaviour of a series of substituted benzoyl­thio­urea derivatives that possess a number of inter­esting properties and reported a thio­ureido ligand in which the two thio­ureido moieties are bridged by a 1,2-phenylene ring (Thiam *et al.*, 2008 ▸). In this paper, we report the synthesis and the characterization of a mol­ecule where the two thio­ureidos are bridged by an ethane-1,2-diyl group.
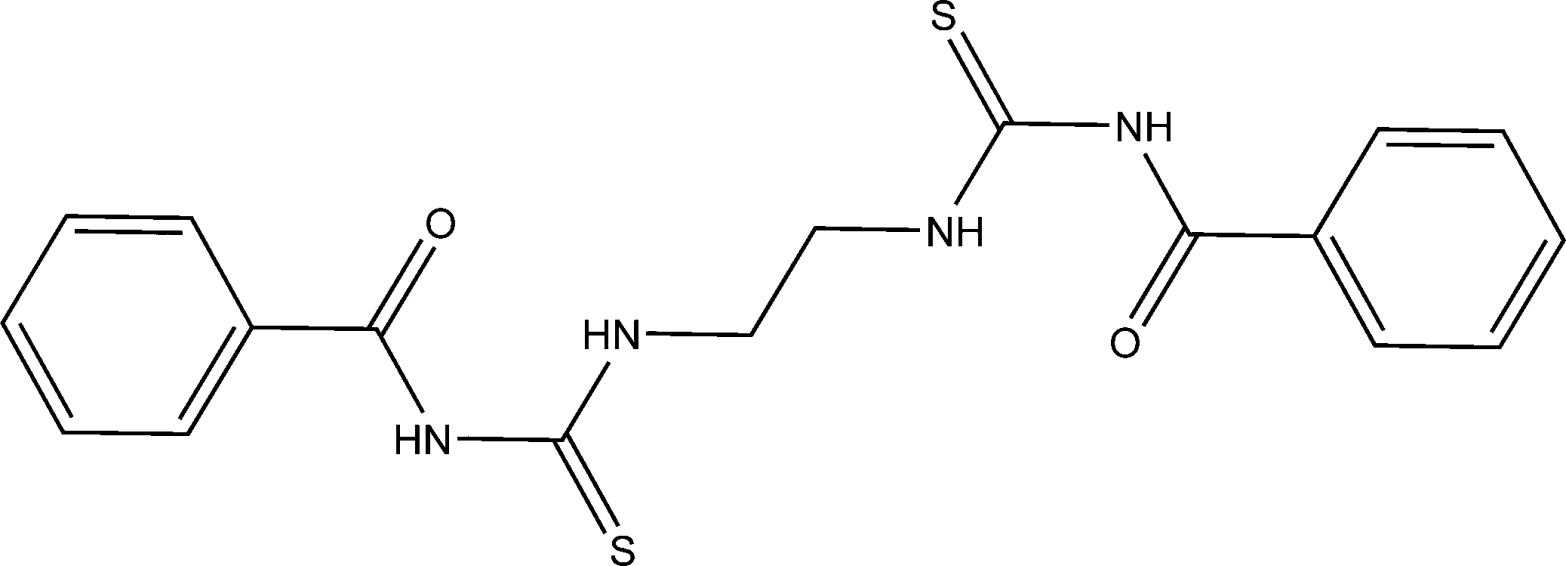


## Structural commentary   

**Figure 1:**
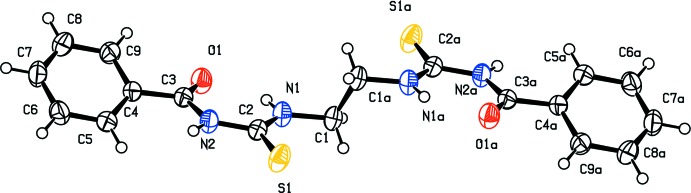
The mol­ecular structure of the title compound, showing the atom-numbering scheme and intra­molecular contacts. Displacement ellipsoids are plotted at the 50% probability level. [Symmetry code: (a) −*x* + 2, −*y* + 1, −*z* + 1]

The asymmetric unit of the title compound is a half-mol­ecule with the other half being generated by an inversion centre located at the mid-point of the C1—C1a bond [Fig. 1 ▸; symmetry code: (a) −*x* + 2, −*y* + 1, −*z* + 1]. The benzoyl groups of each thio­urea subunit are *trans* with respect to the thiono S atoms across the respective C2—N2 bonds. The 1-benzoyl-3-ethyl­thio­urea fragments adopt a *cis* conformation with respect to the thiono S atom across the respective C2—N1 bonds. The S1—C2 [1.6626 (15) Å] and O1—C3 [1.2209 (16) Å] distances indicate that these correspond to double bonds and are comparable to those observed for 1,2-bis­(*N*-benzoyl­thio­ureido)benzene [1.6574 (18) Å for S—C and 1.222 (2) Å for O7—C16] (Thiam *et al.*, 2008 ▸). The C—N bond lengths [1.3744 (17)–1.3971 (17) Å] are in the normal range observed for a single C—N bond. The thio­urea fragments S1/N1/N2/C1/C2 are planar, with a maximum deviation from the least-squares plane of 0.015 (1) Å for the N1 atom. The dihedral angle between this plane and that of the benzene ring (r.m.s. deviation = 0.006 Å) is 26.97 (5)° *versus ca* 34° when the benzene ring is chlorinated (Abusaadiya *et al.*, 2016 ▸). As regularly noticed with carbonyl­urea derivatives, the mol­ecule also forms intra­molecular N1—H1 hydrogen bonds between the carbonyl O and thio­amide H atoms producing *S*(6) rings (N1—H1⋯O1, Table 1 ▸).

## Supra­molecular features   

**Figure 2:**
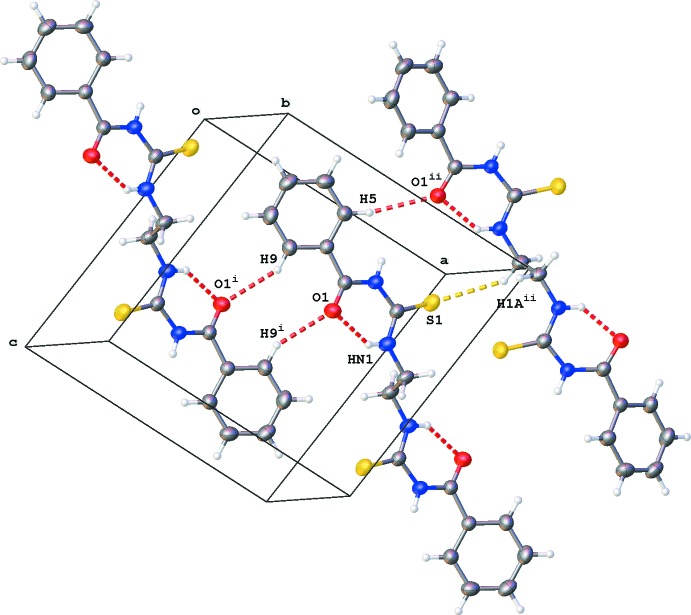
Partial crystal packing of the title compound, showing C—H⋯O (red dashed lines) and C—H⋯S (yellow dashed lines) inter­actions (see Table 1 ▸ for details).

In the crystal, the mol­ecules, which feature an overall Z-form, have both halves roughly parallel to the *ac* plane, whereas the mid-point of the C1—C1a bond lies orthogonally parallel to the (100) plane. Mol­ecular layers running almost parallel to the *ac* plane are formed by inter­molecular C—H⋯O and C—H⋯S inter­actions (Table 1 ▸ and Fig. 2 ▸). These layers stack along the *b* direction. Despite the presence of phenyl rings, no π–π inter­actions are observed in the crystal packing. However, the carbonyl function C3=O1 stacks on phenyl group C4–C9 of a neighbouring layer [O1⋯*Cg*1^iv^ = 3.5543 (14) Å; *Cg*1 is the centroid of ring C4–C9; symmetry code: (iv) −*x* + 1, *y* + 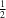, −*z* + 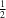].

## Database survey   

Reflecting the inter­est in compounds similar to the title compound, no less than 35 associated structures are included in the Cambridge Structural Database (Version 5.38; Groom *et al.*, 2016 ▸). The match APALEK (Abusaadiya *et al.*, 2016 ▸) is the most similar structure to the title compound, the only difference being the substitution of the phenyl ring on the C3 position by a Cl atom. In both cases, the benzoyl functions of each thio­urea subunit are *trans* with respect to the thiono S atom across the C—N bond. The 1-benzoyl-3-ethyl­thio­urea fragment adopts a *cis* conformation with respect to the thiono S atom across the respective C—N bond. Six structures in which the spacer is different from the spacer in the symmetrical bis­(thio­ureido) mol­ecule studied here appear in the literature. The angles between the phenyl rings are: 63.1° for DAVHOZ (Aydın *et al.*, 2012 ▸), 10.2° for EGUYAH (Sow *et al.*, 2009 ▸), 35.4° for NEWJIL (Light, 2018 ▸), 0.0° for QIXQUK (Ding *et al.*, 2008 ▸), 3.2° for TIFQAD (Oyeka *et al.*, 2018 ▸) and 0.0° for XIQPAP (Dong *et al.*, 2007 ▸). In addition, 23 structures which contain only one arm with a thio­ureido moiety similar to the studied mol­ecules are reported, while the other arm consists of diverse moieties: CIGDAZ (Karipcin *et al.*, 2013 ▸), DELMUD (Ngah *et al.*, 2006 ▸), EYACIQ (Shutalev *et al.*, 2004 ▸), GIHMIV (Haynes *et al.*, 2014 ▸), GIHMOB (Haynes *et al.*, 2014 ▸), IFUZOZ (Hassan *et al.*, 2008*a* ▸,*b* ▸), NIQROV (Yamin & Malik, 2007 ▸), NIQROV01 (Nguyen & Abram, 2008 ▸), POFKIG (Ngah *et al.*, 2014 ▸), QEWHUY (Rakhshani *et al.*, 2018 ▸), RUGKOU (Hassan *et al.*, 2009 ▸), SAFPAT (Wei, 2016 ▸), SITKUC (Yamin *et al.*, 2008 ▸), TADSIB (Zhang *et al.*, 2003 ▸), TADTEY (Yusof & Yamin, 2003 ▸), TIBLEW (Khawar Rauf *et al.*, 2007 ▸). TIHJAW (Yusof *et al.*, 2007 ▸), UNUBAH (Hassan *et al.*, 2011 ▸), WOGTUI (Hassan *et al.*, 2008*a* ▸,*b* ▸), XEBQOM (Adan *et al.*, 2012 ▸), YICDEU (Othman *et al.*, 2007 ▸), YUPYEO (Zheng *et al.*, 2010 ▸) and YUPYEO01 (Khan *et al.*, 2018 ▸).

## Synthesis and crystallization   

All purchased chemicals and solvents were of reagent grade and were used without further purification. Melting points were determined with a Büchi 570 melting-point apparatus and were uncorrected. To a mixture of 7.02 g (72 mmol) of potassium thio­cyanate and 100 ml of acetone was added dropwise a solution of 10.116 g (72 mmol) of benzoyl chloride in 50 ml of acetone. The resulting mixture was stirred under reflux for 1 h and cooled to room temperature. A solution of 2.2 g (36.6 mmol) of 1,2-ethyl­enedi­amine in 20 ml of acetone was added. The yellow solution obtained was stirred at room temperature during 2 h. Hydro­chloric acid (0.1 *N*, 300 ml) was added and a white solid appeared after a few minutes. The compound was filtered off, washed with 3 × 50 ml of water and dried under vacuum. The solid product was washed with water and purified by recrystallization from an ethanol/di­chloro­methane mixture (1:1 *v*/*v*). 12.3 g of the title compound were obtained (yield 88.5%). A small qu­antity of powder was recrystallized from 5 ml of DMF. Colourless single crystals suitable to XRD grew within six days.

## Refinement   

Crystal data, data collection and structure refinement details are summarized in Table 2 ▸. Aromatic H atoms were first located by HFIX and other H atoms were located in the difference Fourier map, positioned geometrically and allowed to ride on their respective parent atoms, with C—H = 0.93 (C_ar_H) or 0.97 Å (CH_2_). The NH H atoms were located in a difference Fourier map and freely refined.

## Supplementary Material

Crystal structure: contains datablock(s) I. DOI: 10.1107/S205698901900495X/vm2216sup1.cif

Structure factors: contains datablock(s) I. DOI: 10.1107/S205698901900495X/vm2216Isup2.hkl

Supporting information file. DOI: 10.1107/S205698901900495X/vm2216Isup3.cml

CCDC reference: 1909438

Additional supporting information:  crystallographic information; 3D view; checkCIF report

## Figures and Tables

**Table 1:** Hydrogen-bond geometry (Å, °)

*D*—H⋯*A*	*D*—H	H⋯*A*	*D*⋯*A*	*D*—H⋯*A*
N1—H*N*1⋯O1	0.86 (2)	1.95 (2)	2.6528 (16)	138 (2)
C5—H5⋯O1^i^	0.93	2.58	3.478 (16)	162
C9—H9⋯O1^ii^	0.93	2.52	3.311 (16)	143
C1—H1*A*⋯S1^iii^	0.97	2.97	3.8375 (16)	150

Symmetry codes: (i) 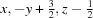; (ii) 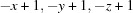; (iii) 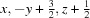.

**Table 2:** Experimental details

Crystal data	
Chemical formula	C_18_H_18_N_4_O_2_S_2_
*M*_r_	386.48
Crystal system, space group	Monoclinic, *P*2_1_/*c*
Temperature (K)	293
*a*, *b*, *c* (Å)	11.2250 (6), 7.2547 (5), 11.1397 (6)
β (°)	100.978 (5)
*V* (Å^3^)	890.55 (9)
*Z*	2
Radiation type	Mo *K*α
μ (mm^−1^)	0.32
Crystal size (mm)	0.36 × 0.14 × 0.11

Data collection	
Diffractometer	XtaLAB AFC12 (RINC): Kappa single
Absorption correction	Multi-scan *CrysAlis PRO* (Rigaku OD, 2018 ▸)
*T*_min_, *T*_max_	0.513, 1.000
No. of measured, independent and observed [*I* > 2σ(*I*)] reflections	7264, 2328, 1942
*R*_int_	0.040
(sin θ/λ)_max_ (Å^−1^)	0.704

Refinement	
*R*[*F* ^2^ > 2σ(*F* ^2^)], *wR*(*F* ^2^), *S*	0.042, 0.121, 1.05
No. of reflections	2325
No. of parameters	124
No. of restraints	2
H-atom treatment	H atoms treated by a mixture of independent and constrained refinement
Δρ_max_, Δρ_min_ (e Å^−3^)	0.36, −0.31

Computer programs: *CrysAlis PRO* (Rigaku OD, 2018 ▸), *SHELXT2014* (Sheldrick, 2015*a* ▸) and *SHELXL2018* (Sheldrick, 2015*b* ▸).

## supplementary crystallographic information

### Crystal data

**Table:**

C_18_H_18_N_4_O_2_S_2_	*F*(000) = 404
*M**_r_* = 386.48	*D*_x_ = 1.441 Mg m^−^^3^
Monoclinic, *P*2_1_/*c*	Mo *K*α radiation, λ = 0.71073 Å
*a* = 11.2250 (6) Å	Cell parameters from 3377 reflections
*b* = 7.2547 (5) Å	θ = 4.7–30.2°
*c* = 11.1397 (6) Å	µ = 0.32 mm^−^^1^
β = 100.978 (5)°	*T* = 293 K
*V* = 890.55 (9) Å^3^	Prism, colourless
*Z* = 2	0.36 × 0.14 × 0.11 mm

### Data collection

**Table:**

XtaLAB AFC12 (RINC): Kappa single diffractometer	2328 independent reflections
Radiation source: micro-focus sealed X-ray tube, Rigaku (Mo)mm03 X-ray Source	1942 reflections with *I* > 2σ(*I*)
Rigaku MaxFlux mirror monochromator	*R*_int_ = 0.040
ω scans	θ_max_ = 30.0°, θ_min_ = 3.7°
Absorption correction: multi-scan CrysAlis PRO (Rigaku OD, 2018)	*h* = −15→14
*T*_min_ = 0.513, *T*_max_ = 1.000	*k* = −10→9
7264 measured reflections	*l* = −15→15

### Refinement

**Table:**

Refinement on *F*^2^	Primary atom site location: dual
Least-squares matrix: full	Secondary atom site location: difference Fourier map
*R*[*F*^2^ > 2σ(*F*^2^)] = 0.042	Hydrogen site location: mixed
*wR*(*F*^2^) = 0.121	H atoms treated by a mixture of independent and constrained refinement
*S* = 1.05	*w* = 1/[σ^2^(*F*_o_^2^) + (0.0683*P*)^2^ + 0.1222*P*] where *P* = (*F*_o_^2^ + 2*F*_c_^2^)/3
2325 reflections	(Δ/σ)_max_ < 0.001
124 parameters	Δρ_max_ = 0.36 e Å^−^^3^
2 restraints	Δρ_min_ = −0.31 e Å^−^^3^

### Special details

**Table:**

Geometry. All esds (except the esd in the dihedral angle between two l.s. planes) are estimated using the full covariance matrix. The cell esds are taken into account individually in the estimation of esds in distances, angles and torsion angles; correlations between esds in cell parameters are only used when they are defined by crystal symmetry. An approximate (isotropic) treatment of cell esds is used for estimating esds involving l.s. planes.

### Fractional atomic coordinates and isotropic or equivalent isotropic displacement parameters (Å^2^)

**Table:**

	*x*	*y*	*z*	*U*_iso_*/*U*_eq_	
N1	0.85736 (10)	0.61198 (19)	0.41958 (11)	0.0399 (3)	
HN1	0.8022 (15)	0.636 (3)	0.4606 (15)	0.048*	
O1	0.62565 (9)	0.65743 (17)	0.43252 (9)	0.0452 (3)	
C1	0.98347 (12)	0.5989 (2)	0.48195 (13)	0.0398 (3)	
H1A	0.996007	0.675814	0.554503	0.048*	
H1AB	1.035920	0.643780	0.428470	0.048*	
S1	0.90847 (3)	0.53047 (7)	0.20190 (4)	0.05100 (17)	
N2	0.69512 (10)	0.59494 (18)	0.25797 (10)	0.0372 (3)	
HN2	0.6711 (17)	0.573 (2)	0.1826 (14)	0.045*	
C2	0.82013 (12)	0.58234 (19)	0.30120 (12)	0.0351 (3)	
C3	0.60474 (11)	0.62787 (19)	0.32261 (12)	0.0332 (3)	
C4	0.47845 (11)	0.61990 (18)	0.25055 (12)	0.0323 (3)	
C5	0.44876 (13)	0.6519 (2)	0.12543 (12)	0.0380 (3)	
H5	0.508903	0.681492	0.081672	0.046*	
C6	0.32849 (14)	0.6391 (2)	0.06642 (14)	0.0446 (3)	
H6	0.308060	0.662390	−0.017073	0.054*	
C7	0.23884 (13)	0.5923 (2)	0.13013 (16)	0.0466 (4)	
H7	0.158787	0.581443	0.089304	0.056*	
C8	0.26809 (14)	0.5617 (2)	0.25436 (16)	0.0469 (4)	
H8	0.207621	0.530909	0.297439	0.056*	
C9	0.38722 (13)	0.5766 (2)	0.31507 (13)	0.0396 (3)	
H9	0.406519	0.557780	0.399116	0.048*	

### Atomic displacement parameters (Å^2^)

**Table:**

	*U*^11^	*U*^22^	*U*^33^	*U*^12^	*U*^13^	*U*^23^
N1	0.0294 (6)	0.0525 (7)	0.0365 (6)	0.0051 (5)	0.0025 (4)	−0.0034 (5)
O1	0.0362 (5)	0.0667 (7)	0.0327 (5)	0.0038 (5)	0.0061 (4)	−0.0008 (5)
C1	0.0288 (6)	0.0468 (8)	0.0405 (7)	−0.0030 (5)	−0.0013 (5)	−0.0052 (6)
S1	0.0316 (2)	0.0798 (3)	0.0436 (2)	−0.00179 (16)	0.01212 (16)	−0.00785 (18)
N2	0.0274 (5)	0.0518 (7)	0.0316 (5)	0.0025 (5)	0.0039 (4)	−0.0014 (5)
C2	0.0282 (6)	0.0395 (7)	0.0371 (6)	−0.0016 (5)	0.0049 (5)	0.0012 (5)
C3	0.0291 (6)	0.0373 (7)	0.0333 (6)	0.0012 (5)	0.0063 (5)	0.0036 (5)
C4	0.0283 (6)	0.0338 (6)	0.0347 (6)	0.0024 (5)	0.0058 (5)	0.0027 (5)
C5	0.0348 (6)	0.0427 (7)	0.0362 (6)	−0.0003 (5)	0.0063 (5)	0.0053 (5)
C6	0.0410 (7)	0.0497 (8)	0.0390 (7)	0.0026 (6)	−0.0030 (6)	0.0041 (6)
C7	0.0285 (6)	0.0490 (9)	0.0586 (9)	0.0040 (6)	−0.0009 (6)	0.0005 (7)
C8	0.0313 (7)	0.0552 (9)	0.0571 (9)	0.0028 (6)	0.0155 (6)	0.0040 (7)
C9	0.0331 (7)	0.0486 (8)	0.0387 (7)	0.0034 (6)	0.0108 (5)	0.0048 (6)

### Geometric parameters (Å, º)

**Table:**

N1—C2	1.3228 (17)	C4—C5	1.3894 (17)
N1—C1	1.4564 (17)	C4—C9	1.3944 (18)
N1—HN1	0.854 (14)	C5—C6	1.388 (2)
O1—C3	1.2209 (16)	C5—H5	0.9300
C1—C1^i^	1.518 (3)	C6—C7	1.380 (2)
C1—H1A	0.9700	C6—H6	0.9300
C1—H1AB	0.9700	C7—C8	1.378 (2)
S1—C2	1.6633 (14)	C7—H7	0.9300
N2—C3	1.3723 (17)	C8—C9	1.383 (2)
N2—C2	1.3971 (16)	C8—H8	0.9300
N2—HN2	0.846 (14)	C9—H9	0.9300
C3—C4	1.4913 (17)		
			
C2—N1—C1	123.98 (12)	C5—C4—C9	119.69 (12)
C2—N1—HN1	116.3 (12)	C5—C4—C3	123.74 (12)
C1—N1—HN1	119.7 (12)	C9—C4—C3	116.58 (12)
N1—C1—C1^i^	110.75 (14)	C6—C5—C4	119.34 (13)
N1—C1—H1A	109.5	C6—C5—H5	120.3
C1^i^—C1—H1A	109.5	C4—C5—H5	120.3
N1—C1—H1AB	109.5	C7—C6—C5	120.78 (14)
C1^i^—C1—H1AB	109.5	C7—C6—H6	119.6
H1A—C1—H1AB	108.1	C5—C6—H6	119.6
C3—N2—C2	128.67 (11)	C8—C7—C6	119.91 (13)
C3—N2—HN2	115.1 (13)	C8—C7—H7	120.0
C2—N2—HN2	116.1 (13)	C6—C7—H7	120.0
N1—C2—N2	116.03 (12)	C7—C8—C9	120.11 (14)
N1—C2—S1	125.77 (10)	C7—C8—H8	119.9
N2—C2—S1	118.19 (10)	C9—C8—H8	119.9
O1—C3—N2	122.46 (12)	C8—C9—C4	120.15 (14)
O1—C3—C4	121.88 (12)	C8—C9—H9	119.9
N2—C3—C4	115.64 (11)	C4—C9—H9	119.9
			
C2—N1—C1—C1^i^	−84.6 (2)	N2—C3—C4—C9	154.33 (13)
C1—N1—C2—N2	177.90 (13)	C9—C4—C5—C6	−0.4 (2)
C1—N1—C2—S1	−1.5 (2)	C3—C4—C5—C6	179.28 (13)
C3—N2—C2—N1	−2.5 (2)	C4—C5—C6—C7	−1.1 (2)
C3—N2—C2—S1	177.01 (12)	C5—C6—C7—C8	1.5 (3)
C2—N2—C3—O1	2.4 (2)	C6—C7—C8—C9	−0.4 (3)
C2—N2—C3—C4	−175.85 (13)	C7—C8—C9—C4	−1.0 (2)
O1—C3—C4—C5	156.36 (14)	C5—C4—C9—C8	1.4 (2)
N2—C3—C4—C5	−25.33 (19)	C3—C4—C9—C8	−178.28 (14)
O1—C3—C4—C9	−23.98 (19)		

Symmetry code: (i) −*x*+2, −*y*+1, −*z*+1.

### Hydrogen-bond geometry (Å, º)

**Table:**

*D*—H···*A*	*D*—H	H···*A*	*D*···*A*	*D*—H···*A*
N1—H*N*1···O1	0.86 (2)	1.95 (2)	2.6528 (16)	138 (2)
C5—H5···O1^ii^	0.93	2.58	3.478 (16)	162
C9—H9···O1^iii^	0.93	2.52	3.311 (16)	143
C1—H1*A*···S1^iv^	0.97	2.97	3.8375 (16)	150

Symmetry codes: (ii) *x*, −*y*+3/2, *z*−1/2; (iii) −*x*+1, −*y*+1, −*z*+1; (iv) *x*, −*y*+3/2, *z*+1/2.
